# Acute shortening and re-lengthening (ASRL) procedure combined with RhBMP-2 to treat femur non-union with leg length discrepancy: a case report

**DOI:** 10.1097/RC9.0000000000000431

**Published:** 2026-04-01

**Authors:** Asep Santoso, Firda Nur Rahmi, Muhammad Riyadli, Fanny Indra Warman, Bagus Iman Brilianto, Iwan Budiwan Anwar

**Affiliations:** Department of Orthopaedic and Traumatology, Prof. Dr. R. Soeharso Orthopaedic Hospital – Universitas Sebelas Maret, Sukoharjo, Central Java, Indonesia

**Keywords:** acute shortening and re-lengthening, femoral non-union, leg length discrepancy, monorail fixator, rhBMP-2

## Abstract

**Background:**

Femoral shaft non-union complicated by leg length discrepancy (LLD) and segmental bone loss remains a significant reconstructive challenge. Conventional bone transport techniques may involve long external fixation times, multiple surgeries, and soft-tissue morbidity. The acute shortening and re-lengthening (ASRL) method offers an alternative by achieving immediate bone contact and tissue closure, followed by gradual restoration of length. The adjunctive use of recombinant human bone morphogenetic protein-2 (rhBMP-2) may enhance osteogenesis at the docking and regenerate sites.

**Case presentation:**

We present a 24-year-old male patient with a femoral non-union and a 6 cm leg length discrepancy following multiple failed surgeries and hardware removal. The operative strategy comprised thorough debridement of the non-union site, acute shortening of the femur for direct end-to-end contact, application of rhBMP-2 at the docking interface, and placement of a monorail external fixator. A proximal femoral corticotomy was then performed for gradual distraction at 1 mm/day after a 10-day latency. Full-length restoration, radiographic union, and pain-free weight-bearing were achieved at 12 months. No major complications occurred.

**Clinical discussion:**

The ASRL technique, combined with rhBMP-2 on docking site, may represent a viable biomechanical-biological strategy for complex femoral non-union with LLD.

**Conclusions:**

The combined mechanical-biological strategy of ASRL with *rhBMP-2* augmentation at docking site may offer a viable method for addressing femoral shaft non-union with leg length discrepancy.

## Introduction

Non-union of the femoral shaft remains a difficult problem in orthopedic trauma surgery. When compounded by segmental bone loss and leg length discrepancy (LLD), functional impairment, prolonged treatment and risk of complications rise significantly. In long-bone defects, the traditional paradigm of bone transport via circular or rail external frames has been well established; however, the extended duration of fixation, high incidence of pin-tract infection, joint stiffness, and patient discomfort remain major drawbacks^[^1,2^]^. The acute shortening and re-lengthening (ASRL) technique used to overcome some of these limitations. By acutely shortening the leg to close the bone gap and restore bone continuity, followed by a corticotomy and gradual re-lengthening, the method permits earlier soft-tissue closure and reduces the period during which the bone and soft-tissue environment remain compromised^[^3^]^.

HIGHLIGHTS

- Acute shortening and re-lengthening (ASRL) is an effective technique for managing femoral non-union with significant bone loss and leg length discrepancy.
- Combination with recombinant human bone morphogenetic protein-2 (rhBMP-2) promotes osteogenesis and accelerates bone healing in challenging non-union cases.
- The procedure allows early bone contact, improved stability, and faster consolidation compared to conventional bone transport methods.
- Use of monorail external fixator facilitates precise lengthening control and minimizes soft-tissue complications.
- This case demonstrates successful bone union and restoration of limb length without major complications, highlighting the synergistic role of ASRL and rhBMP-2 in complex femoral reconstruction.

Biologically, non-unions and segmental defects often coincide with compromised local biology – reduced vascularity, fibrotic soft tissues, and diminished osteogenic stimuli. The “diamond concept” of bone healing emphasizes four key elements: osteogenic cells, scaffolding, vascularity, and mechanical stability^[^4^]^. Recombinant human BMP-2 (rhBMP-2) has been used as a biological stimulus in non-union surgery, particularly in the femur. In a series of femoral non-unions treated with rhBMP-2, union was achieved in four of five pediatric cases, with a mean time to union of 12 months^[^5^]^. Although BMP-2 use in femoral non-unions is supported by emerging evidence, its application specifically in concert with ASRL in femoral cases is rarely reported. We hence report a case of femoral shaft non-union with LLD treated with ASRL combined with rhBMP-2, documenting surgical technique, radiographic and functional outcome. No artificial intelligence tools were used in the preparation, writing, editing, or analysis of this manuscript. This work has been reported in line with the SCARE 2025 criteria^[^6^]^.

## Case presentation

**Figure 1.**
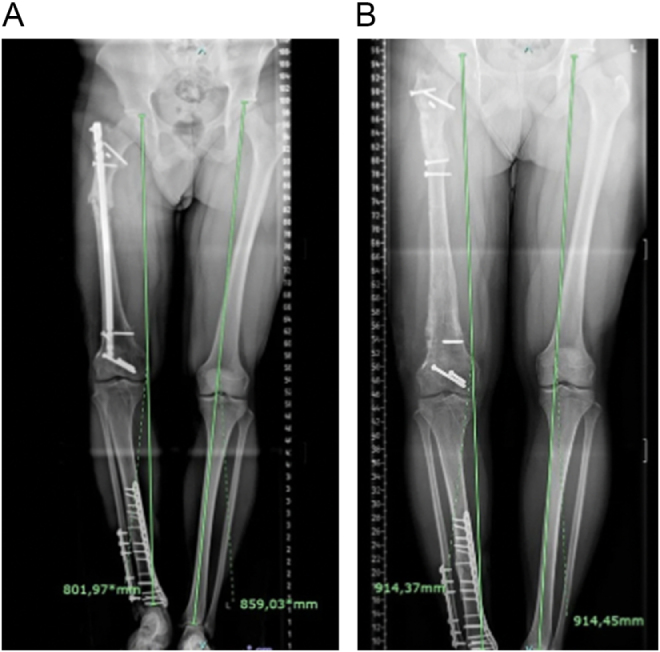
(A) Pre-operative full leg radiograph, (B) Final full leg radiograph after removal of external fixator at18 months after first surgery (residual varus mechanical alignment is present).

A 24-year-old male college student, presented with persistent pain and instability of the right thigh, together with a 6 cm shortening of the right lower leg. His history included a high-energy motor-vehicle femoral shaft fracture 18 months prior, treated initially with intramedullary nailing, which subsequently failed. This was followed by hardware removal, exchange nailing, and bone grafting, all of which resulted in non-union at the mid-shaft and shortening, with limited knee flexion (0–90°) and gait impairment. Clinical examination revealed tenderness at the non-union site, leg length discrepancy (LLD) of 6 cm (measured by block method), and no active infection (normal C-reactive protein/white blood cells). Full leg lower extremity radiographs demonstrated atrophic non-union with bone loss, anatomical femoral bone comparison between right and left side showed LLD of 4.5 cm with varus mechanical malalignment of 5°of lower extremity. This resulted a total of 6 cm radiologic LLD (Fig. 1A). Clinical evaluation for the sagital and rotational deformity was performed and the result was negative.

Given the failed prior interventions, bone loss, shortening and soft-tissue concerns, we planned an ASRL procedure with rhBMP-2 augmentation on docking site. Under general anesthesia and prophylactic antibiotics, first removal of femoral nail was performed, followed with exposure of the non-union site via a lateral approach. All fibrous tissue and sclerotic bone ends were debrided until viable bleeding cortex was obtained. Acute shortening was performed to obtain end-to-end contact, align the mechanical axis, and allow soft-tissue closure. A monorail external fixator was applied, spanning from proximal femur to distal femur with three Schanz screws proximally and three distally. At the docking site we applied 2 mg of *rhBMP-2* (Novosis, CGBio, Daewoong, Korea) between the bone ends. A distal metaphyseal corticotomy was then created via percutaneous technique, and fixator distraction components applied. Post-operative protocol included a latency period of 10 days, followed by distraction at 1 mm/day in four increments of 0.25 mm.

**Figure 2.**
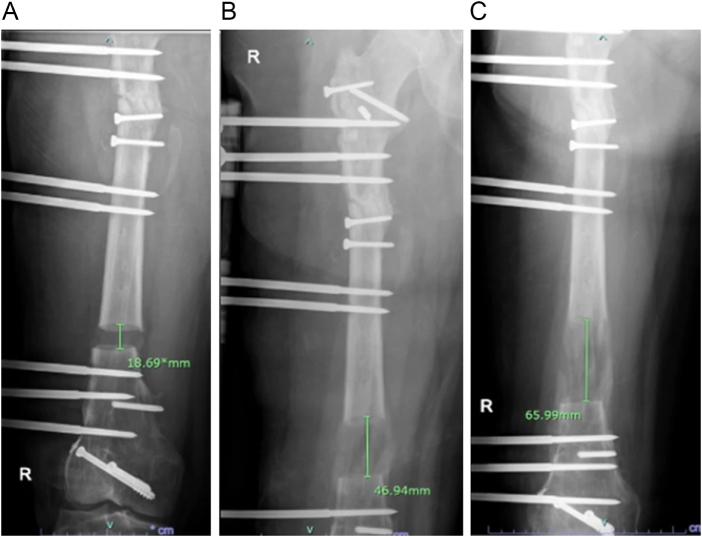
(A) Radiograph at 1-month post-op; (B) Radiograph at 3 months post-op; (C) Radiograph at 5 months post-op.

**Figure 3.**
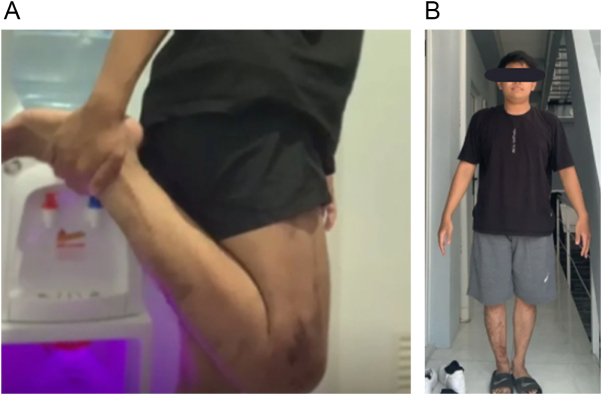
Clinical outcomes. (A) Satisfactory range of motion of the knee at 2 year-follow-up, (B) Equal leg length.

Weight-bearing as tolerated with crutches was initiated. Radiographic assessments were performed bimonthly. At 2 months the regenerate zone showed early callus formation with progressive consolidation at 3 and 5 months. At 5 months, the overall 6.5 cm bone distraction was achieved which confirmed radiographically (Fig. 2A-C). Although, residual varus mechanical alignment was noted on final radiograph (Fig. 1B), the final clinical leg length was equal (Fig. 3B). This is possibly due to the extra bone regenerate of 0.5 cm compensating the residual mechanical varus alignment. At 9 months, consolidation of the docking site and regenerate zone was evident with bridging callus on three of four cortices with clinical knee stiffness (flexion 0–90^o^). At 12 months, the external fixator was removed along with surgical release of soft tissue and knee joint manipulation to achieve knee full flexion. Final full-length radiographs after external fixator removal showed restoration of leg length and final alignment of lower extremity (Fig. 1B).

The patient achieved full weight-bearing without assistive devices, pain-free ambulation, and knee flexion improved to 0–120°. No major complications (neurovascular injury, wound dehiscence, deep infection) were encountered. Minor pin-tract infection occurred at several Schanz screw site at month 3, managed successfully with oral antibiotics. At 15-month follow-up, the leg remained equal in length, alignment was maintained with residual varus mechanical alignment, and the patient returned to his previous work with satisfactory knee range of motion (Fig. 3A). This improvement in mobility allowed the patient to resume schooling, marking a considerable positive impact on his social life.

## Discussion

The management of femoral non-union with segmental defect and leg length discrepancy requires optimization of both mechanical and biological environments. The ASRL technique relies on the advantages of immediate end-to-end bone contact, followed by gradual restoration of length via distraction osteogenesis. In a comparative study of tibia infected non-unions, ASRL achieved significantly reduced external fixation duration and index compared with bone transport, without compromising bone or functional results^[^2^]^. Biomechanically, acute shortening brings the bone ends into contact, which facilitates the biologic healing cascade by reducing gap size, improving stability, and enhancing load transfer. It also permits primary soft-tissue closure, which reduces infection risk and improves vascular milieu. Once the bone ends are healed or stable, the corticotomy regenerate provides endothelial and osteoblastic driven new bone in a distraction environment^[^2,7^]^.

The combination therefore addresses both bone defect closure and length restoration in a staged fashion. The adjunctive use of rhBMP-2 in non-union surgery brings a biological advantage, particularly in compromised environments. rhBMP-2 is an osteo-inductive protein that drives mesenchymal stem cell differentiation into osteoblasts, stimulates angiogenesis, and enhances callus formation^[^8^]^. Clinical studies in femoral non-unions have reported favorable union rates with rhBMP-2 uses. A large long-bone non-union cohort found that union rates were significantly higher with the use of BMP-2. A previous study reported that 33 femoral non-unions treated using rhBMP-2 were healed significantly faster than control group (85% vs 44%, respectively). Similar results also reported on tibia non-unions cases, 25 out of 27 (93%) healed with a median of 9 months after rhBMP-2 application with significantly higher union rate compared to the no-BMP group (33%)^[^9^]^. These data suggest that while autograft remains the gold standard, the application of rhBMP-2 may be a valuable adjunct in high-risk non-unions, such as the present case, where the local biology is compromised and the mechanical environment is being acutely altered, thereby directly addressing the “biological” pillar of the diamond concept. In the present case, the synergy of ASRL method and rhBMP-2 application is notable. The acute shortening closed the bone defect, allowed soft-tissue restoration, and facilitated early mechanical stability. The application of rhBMP-2 at the docking site likely enhanced the osteogenic activity where bone–bone contact had been achieved. The subsequent corticotomy and distraction restored the clinical leg length discrepancy with regenerate formation.

The use of a monorail fixator simplified frame management compared to circular systems, improving patient tolerance. However, the technique is not without limitations. Acute shortening beyond safety thresholds (often >4–6 cm) may risk neurovascular compromise, soft-tissue bunching, venous congestion, and leg-length inequality^[^2,3,7^]^. Careful intraoperative assessment of leg perfusion and staged shortening may be required for larger defects. Furthermore, while rhBMP-2 use is promising, evidence remains limited, particularly in femoral ASRL contexts. Finally, patient compliance, frame management, and meticulous follow-up are critical. The external fixation index (EFI) remains a useful metric: in the aforementioned femoral series ASRL achieved 31.8 days/cm compared to 48.7 days/cm for bone transport^[^10^]^. In our case, a lengthening of 6.5 cm with fixator removal at 12 months yields an EFI of around 56 days/cm, which is higher. This higher index is likely attributable to the complexity of the case, which included multiple failed prior surgeries, poor soft tissue envelope, and the need for a concurrent knee manipulation and soft tissue release at the time of frame removal, factors which can prolong the overall treatment and fixation time. Another important issue is regarding the economic aspect of application of rhBMP-2 which may give additional economic burden, especially in developing/low-income country.

## Conclusions

The combined mechanical-biological strategy of ASRL with *rhBMP-2* augmentation at docking site may offer a viable method for addressing femoral shaft non-union with leg length discrepancy. In this case, the method achieved full union, restoration of leg length, and excellent functional recovery with minimal complications. While the results are encouraging, further prospective studies and longer-term follow-up are required to establish optimal patient selection, shortening/lengthening thresholds, adjunctive biological use, and frame management protocols.

## Data Availability

The data that support the findings of this study are available from the corresponding author upon resonable request.
